# Case Report: Micro-RNAs in Plasma From Bilateral Inferior Petrosal Sinus Sampling and Peripheral Blood From Corticotroph Pituitary Neuroendocrine Tumors

**DOI:** 10.3389/fendo.2022.748152

**Published:** 2022-04-22

**Authors:** Helvijs Niedra, Raitis Peculis, Ilze Konrade, Inga Balcere, Mihails Romanovs, Liva Steina, Janis Stukens, Jelizaveta Sokolovska, Janis Klovins, Vita Rovite

**Affiliations:** ^1^ Department of Molecular and Functional Genomics, Latvian Biomedical Research and Study Centre, Riga, Latvia; ^2^ Department of Endocrinology, Riga East Clinical University Hospital, Riga, Latvia; ^3^ Department of Internal Diseases, Riga Stradins University, Riga, Latvia; ^4^ Department of Neurosurgery, Pauls Stradins Clinical University Hospital, Riga, Latvia; ^5^ University of Latvia Faculty of Medicine, Riga, Latvia

**Keywords:** micro-RNA differential expression, bilateral inferior petrosal sinus sampling, circulating plasma micro-RNAs, corticotroph pituitary neuroendocrine tumor, Cushing’s disease

## Abstract

**Objective:**

Circulating miRNAs are found in bodily fluids including plasma and can serve as biomarkers for diseases. The aim of this study was to provide the first insight into the landscape of circulating miRNAs in close proximity to the adrenocorticotropic hormone (ACTH) secreting PitNET. To achieve this objective next-generation sequencing of miRNAs in plasma from bilateral inferior petrosal sinus sampling (BIPSS) - a gold standard in diagnosing ACTH-secreting PitNETs was carried out and selected miRNA candidates were further tested by RT-qPCR in independent patient cohorts.

**Methods:**

Sinistral (left) and dextral (right) BIPSS blood samples of the patient were collected in three time points: before the administration of corticotropin-releasing hormone, 5 and 15 minutes after stimulation. In differential expression analysis, sinistral plasma was compared with dextral. The selected miRNA candidates were tested in plasma by RT-qPCR in two patient groups: 1) in five ACTH secreting PitNET patients with plasma samples taken before and 24 hours after surgery, 2) in 12 ACTH secreting PitNET patients vs. 9 non-functioning PitNET patients.

**Results:**

BIPSS concluded that the highest amount of ACTH was released in the sinistral side at the 5^th^ minute mark indicating a presence of a tumor. The highest amount of differentially expressed miRNAs was observed 5 minutes after stimulation (20 upregulated, 14 downregulated). At the 5^th^ minute mark in sinistral plasma, two miRNAs were identified: hsa-miR-7-5p and hsa-miR-375-3p that were highly upregulated compared to other BIPSS samples and peripheral plasma samples. Further testing by qPCR revealed significant reduction of miR-7-5p in plasma 24 hours after surgery and upregulation in plasma of ACTH secreting PitNET patients compared to non-functioning PitNET patients (P =0.0013).

**Conclusions:**

By stimulating the ACTH secreting PitNET with CRH a rapid increase of two miRNAs (hsa-mir-7-5p, hsa-mir-375-3p) and ACTH can be observed in sinistral inferior petrosal (tumor side). A decrease of miR-7-5p in plasma after surgery and upregulation in plasma of ACTH secreting PitNET patients was discovered implying that further studies of this miRNA as diagnostic marker is needed.

## Introduction

ACTH-secreting PitNETs are responsible for the majority of ACTH-dependent Cushing’s syndrome (CS) cases (75 - 80%) while the other 15% - 20% can be classified as CS with an ectopic origin (1). It is also estimated that only 6% - 9% of ACTH-dependent CS cases can be attributed to large (>10 mm diameter) ACTH-secreting tumors (2, 3). To prescribe appropriate treatment it is vital to correctly assess the main cause of ACTH-dependent CS and as the first step patients are required to undergo magnetic resonance imaging of the pituitary gland (MRI) (4). However, in the study by Invitti et al. which investigated PitNETs cases that are less than 5 mm in diameter, it was concluded that only 53% of the MRI scans yield conclusive results (5). In a CS scenario when the results of MRI scan are inconclusive in confirming the presence and location of a tumor the patients may be required to undergo a bilateral inferior petrosal sinus sampling (BIPSS) procedure (4). BIPSS is a gold-standard procedure for distinguishing between pituitary and non-pituitary sources of ACTH-hypersecretion. It involves simultaneous catheterization of inferior petrosal sinuses (IPSs) which directly drain the pituitary gland. The catheterization itself is done *via* the transjugular approach and after the position of both catheters is adjusted an intravenous administration of desmopressin or corticotropin-releasing hormone (CRH) is performed. This stimulates the corticotrophs of the pituitary gland and PitNET to release ACTH. After the stimulation of corticotrophs, blood samples are taken directly from IPS, and concentrations of ACTH are measured. Depending on the setting this diagnostic approach has up to 100% sensitivity and specificity in identifying whether the source of ACTH hypersecretion is the PitNET and on which side (sinistral or dextral) the tumor is located (6, 7).

miRNAs are a class of small non-coding RNAs with an average length of 22 nucleotides. miRNAs play a vital role in post-transcriptional gene expression regulation by binding to the 3’ UTR region of target mRNAs and up to 60% of human protein-coding genes contain conserved targets for miRNA-mRNA interaction (8–10). For this reason, they have been extensively studied in the context of cancer development, progression, diagnostics, and therapy with a vast amount of miRNAs showing promising results as both tissue biopsy and liquid biopsy biomarkers (11, 12). Regarding PitNETs miRNAs also have been studied both in tumor tissue and as blood-based biomarkers. In tissue, it has been shown that upregulation of miR-34a and miR-107 is directly associated with downregulation of AIP expression, one of the most studied genes in familial isolated PitNETs and a proposed tumor-suppressor (13–15). In ACTH-secreting PitNETs miR-26a is significantly upregulated compared to normal pituitary tissues with its direct target being cell cycle regulation associated protein kinase PRKCD (16).

As of today, there is a limited amount of information on circulating extracellular vesicle-associated miRNAs in the blood plasma of PitNET patients. Nemeth K. et al. were the first ones to provide insight into potential plasma-derived miRNA biomarkers for PitNET with miR-143-3p emerging as a candidate biomarker for post-surgery monitoring of non-functioning follicle stimulating hormone and luteinizing hormone immunopositive (NF FSH/LH+) PitNET patients (17). Another study showed that plasma miRNAs: miR-16-5p, miR-145-5p, and let-7g-5p are upregulated in CS patients with ACTH-secreting PitNETs versus CS patients with ectopic cause (18).

Detecting PitNET specific miRNAs in plasma samples remains a challenging task due to their high dilution. In this study, we had a unique opportunity to study PitNET-derived circulating extracellular vesicle (EVs) associated miRNAs in close proximity to the tumor itself. Using next generation sequencing (NGS) we analyzed miRNA contents in plasma from IPSs and in plasma from peripheral venous blood (PVB) of single BIPSS patient case. We hypothesized that there would be detectable differences in expression of circulating EVs bound miRNAs between sinistral and dextral sides of IPSs as well as differences between BIPSS and PVB plasma. To confirm this hypothesis, we collected plasma samples from the BIPSS procedure in three separate time points and compared the miRNA fractions found in PVB using NGS analysis. The candidate hits were further validated in PVB plasma of two independent patient cohorts and their expression was also checked within tumor tissue using RT-qPCR approach.

## Materials and Methods

### BIPSS Case Patient Recruitment

The BIPSS patient was recruited from Riga East Clinical University Hospital where she underwent BIPSS in 2018. Before the acquisition of biological samples and clinical data two written informed consents were obtained from the patient: consent for the use and storage of biological material in the national biobank Genome Database of Latvian population (LGDB) for health and hereditary research and PitNET research specific consent (19). The use of patients’ samples and data in this study was approved by the Central Medical Ethics Committee of Latvia (protocol: 22.03.07/A7 and 2/18-02-21).

BIPSS was done by an experienced team of interventional radiologists and endocrinologists using a percutaneous femoral vein approach. After obtaining basal samples, an intravenous injection of 100mcg human CRH (hCRH) was administered as a bolus, and post-stimulation samples were obtained at 0, 3, 5, 10, and 15 minutes. All samples were immediately collected in tubes containing EDTA and immediately centrifugated. During the BIPSS procedure, the blood samples for miRNA extractions were also collected in EDTA tubes in three main steps (**Supplementary Figure 1**). Two months after BIPSS patient underwent transsphenoidal surgery during which the medical staff collected PVB samples before surgery and 24 hours after surgery.

### Validation Cohort Patient Recruitment and Characteristics

To additionally test the plasma miRNA markers discovered in analysis of BIPSS patient we recruited two additional groups of PitNET patients (26 patients in total) from Riga East Clinical University Hospital and Pauls Stradins Clinical University Hospital. The 1^st^ patient group included five ACTH secreting PitNET patients (OP1 – OP5) who underwent transsphenoidal resection of the PitNET (**Table 1**). For these patients PVB samples were collected before and 24 hours after resection. We also collected tumor tissue samples from these five patients which were used to confirm the expression of selected markers within the PitNET tissues. Prior to the surgery three out of five patients had received medication in form of Fluconazole. Successful remission after surgery was observed for three out of five patients. Details on each patient regarding pre-surgical medical treatment and post-surgical remission status can be viewed in **Table 1**. The 2^nd^ patient group included 12 ACTH (ACTH1 – ACTH12) secreting PitNET patients and 9 non-fucntioning (NF) PitNET patients (NF1 – NF9). According to 2017 WHO PitNET classification guidelines (20) our immunohistochemistry findings confirmed that five out of 9 NF PitNET patients had NF FSH/LH+ PitNETs while the remaining 4 had NF immunonegative tumors. Detailed information on these patients’ characteristics is available on **Supplementary Table 1**. For these 21 patients PVB samples were collected and were used to evaluate the expression candidate miRNAs between ACTH secreting PitNETs and NF PitNETs.

**Table 1:** Group of patients with pre and postoperative plasma samples for evaluation by qPCR.

Patient	Sex	PitNET type	Tumor size	Age of diagnosis	Medical treatment	Remission after surgery	Available tumor tissue sample
OP1	F	ACTH secreting	Mi	29	Fluconazole	No, unsuccessful surgery	FFPE
OP2	F	ACTH secreting	Ma	60	Fluconazole	No, relapse after 6 months	Fresh frozen
OP3	M	ACTH secreting	Ma	58	Fluconazole	Yes	Fresh frozen
OP4	F	ACTH secreting	Mi	48	No medical treatment	Yes	FFPE
OP5	F	ACTH secreting	Ma	66	No medical treatment	Yes	Fresh frozen

ACTH, adrenocorticotropic hormone; F, female; M, male; Mi, microadenoma; Ma, macroadenoma; FFPE, formalin fixed paraffin embedded.

### Blood Sample Processing and Total RNA Extraction

All EDTA collected blood samples used for miRNA isolation were stored at RT until centrifugation. The plasma was separated from whole blood directly after sample collection by two-step centrifugation: 2000 RPM for 10 minutes at RT and 4000 RPM for 10 minutes at RT. After centrifugation, the plasma samples were aliquoted at 1ml and stored at -80°C. Plasma located EVs were isolated, and their RNA contents were extracted from 0.5 mL plasma samples using exoRNeasy Midi kit (Qiagen, Germany) according to the manufacturer’s instructions. During the extraction 52 synthetic miRNA spike-ins from QIAseq miRNA Library QC qPCR Assay Kit (Qiagen, Germany) were added according to the manufacturer’s instructions before the RNA purification part. Extracted RNA samples were stored under -80°C. Total RNA from fresh frozen samples was extracted using AllPrep DNA/RNA/miRNA Universal Kit (Qiagen, Germany) according to the manufacturer’s instructions. As for the total RNA extraction from FFPE samples RNeasy FFPE kit (Qiagen, Germany) was used according to the manufacturer’s instructions. Concentrations of the extracted RNA were measured with Qubit 2.0 (Thermo Fisher, USA) miRNA library preparation and NGS

To determine the validity of extracted RNA samples prior to downstream NGS analysis we carried out qPCR on ViiA™ 7 (Applied Biosystems, USA) using QIAseq miRNA Library QC qPCR Assay Kit. qPCR reactions were set up following the manufacturer’s instructions. QIAseq miRNA Library QC qPCR Assay Kit included 8 assays which allowed us to assess the following conditions: extraction efficiency and uniformity (UniSp100 and UniSp101, **Supplementary Figure 2**), cDNA synthesis efficiency (UniSp6, **Supplementary Figure 3**), evaluation of hemolysis (miR-23a-3p and miR-451a, **Supplementary Figure 4**), presence of constitutively expressed plasma miRNAs (miR-103a-3p, miR-191-5p, miR-30c-5p, **Supplementary Figure 5**).

All extracted RNA samples passed quality standards underwent miRNA library preparation using Small RNA-Seq Library Prep Kit (Lexogen, Austria) according to the manufacturer’s instructions. To avoid the contamination of adapter dimers and RNAs that exceed the range of small ncRNAs we employed BluePippin electrophoresis (Sage Science, USA) for the size selection step. According to Lexogen’s user manual fragments around 140 – 150 bp indicate a small ncRNA library so the target range for BluePippin electrophoresis was set 125 – 160bp as it was found to be the most optimal for yielding purified library. The results after BluePippin were visualized using High Sensitivity DNA Chip and Kit on Agilent 2100 bioanalyzer (Agilent Technologies, USA) and concentrations were measured using Qubit 2.0 (Thermo Fisher, USA). Libraries were sequenced on the MiSeq NGS system (Illumina, USA) with a target range of 4 million reads per sample. During extraction, library preparation, and sequencing the samples were processed in two batches (**Supplementary Table 2**). NGS data analysis and differential expression analysis

The NGS data were analyzed using CLC genomics workbench (v20.0.4.) (Qiagen, Germany). Adapters were trimmed from the 3’ end and any reads with the quality score < 0.02 were discarded. Regarding the length-based trimming, any reads that did not fall into the range of 15 - 55 nucleotides were discarded (**Supplementary Figure 6**). For miRNA and spike-in quantification, the reads to were aligned miRBase (v22) and qiaseq_mirna (v1) spike-ins dataset. For alignment, maximum allowed mismatches were set to 2 and for length-based isomiRs maximum upstream/downstream bases were set to 2. Allowed sequence length was set from 18 to 25 bp (size of mature miRNA). 52 spike-ins which were added during RNA extraction were annotated and counted using qiaseq_mirna_v1 spike-in reference. Counts were correlated between samples in R 4.1.2. using Pearson’s correlation and R^2^ values matrix was created to evaluate sample-to-sample similarities in spike-in counts (**Supplementary Table 5**). According to QIAseq miRNA Library QC Spike-ins kit user manual R^2^ >= 0.95 represents a good correlation between samples.

Differential expression analysis was performed in CLC Genomics Workbench (v20.0.4). The methodology is based on negative binomial distribution and uses trimmed mean of m-values (TMM) as a normalization method. Count values are output as counts per million (CPM). For BIPSS samples differential expression analysis we compared the dextral side with the sinistral side in three separate time points: 0 minutes (before administration of CRH), 5 minutes after stimulation, and 15 minutes after stimulation. For PVB samples we compared before BIPSS with after BIPSS and before surgery with after surgery. We chose to represent results that met the following criteria: Bonferroni P < 0,05. Fold changes were represented as Log2 fold change (Log2FC).

### Validation of Candidate MiRNAs by Quantitative Reverse Transcription Polymerase Chain Reaction (RT-qPCR)

RT-qPCR for miRNA testing in pre and postoperative patient group and in ACTH vs. NF PitNET patient group was done in two steps. First one involved cDNA synthesis using miRCURY LNA RT kit (Qiagen, Germany) which was followed by SYBR green based qPCR reaction set up using miRCURY LNA SYBR Green PCR kit (Qiagen, Germany) according to the manufacturer’s instructions. Predesigned locked nucleic acid (LNA) primers of miR-375-3p and miR-7-5p were ordered online using GeneGlobe resources (Qiagen, Germany). The sequences of the primers are unavailable due to the manufacturer’s policies. Reactions for each sample were set up in triplicates. As a housekeeping gene for data normalization, we chose to amplify and use synthetic miRNA spike-ins UniSp100 and UniSp101 which were added during EV associated RNA extraction from plasma samples. qPCR was done of ViiA 7 cycler (ThermoFisher, USA) and the results were analysed using ΔΔCt method (21). Statistical analysis was done on R 4.1.2. For the pre- and postoperative plasma comparison the two groups were compared using paired t-test and Wilcoxon rank sum test depending on the distribution of the data. The normality of the data was checked using Shapiro-Wilk normality test. The data further were imported in GraphPad Prism 9.2.0 for visualization (GraphPad Software Inc., USA).

The expression of candidate hits was also checked in fresh frozen tissue and formalin-fixed paraffin-embedded tissue samples (FFPE) using the RT-qPCR approach. For miRCURY LNA RT kit reverse transcription reaction a 6 ng of total RNA was used for each sample. qPCR was done using miRCURY LNA SYBR Green PCR kit according to the manufacturer’s instructions.

## Results

### BIPSS Patient Clinical Characteristics

**Figure 1:**
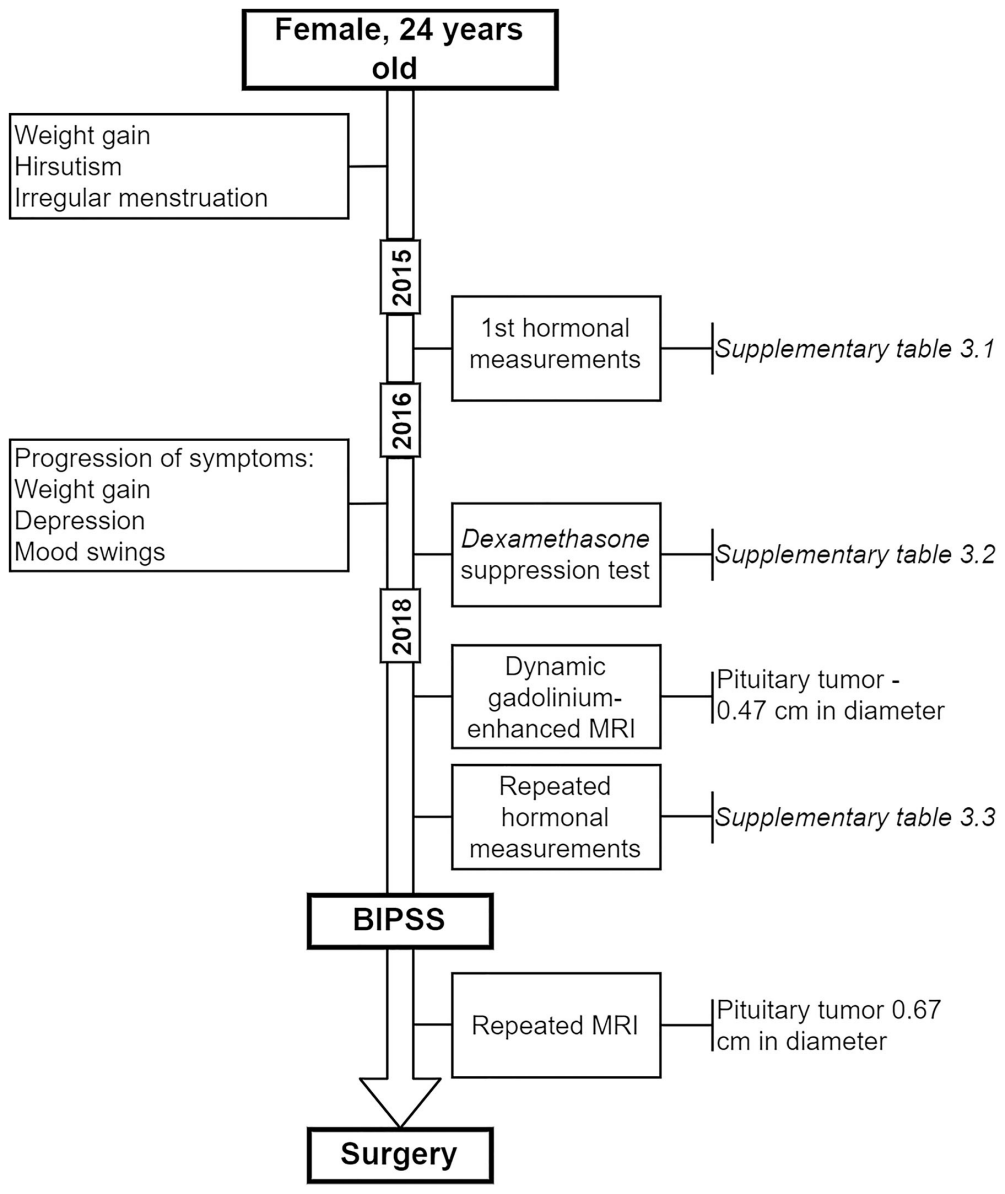
Timeline of BIPSS patient.

The enrolled patient (female) was 24 years old when the first symptoms of an irregular menstrual cycle, hirsutism, and weight gain developed (**Figure 1**). The first hormonal measurements were done in late 2015 (**Supplementary Table 3.1**) which didn’t show any signs of hypercortisolemia (24h urinary cortisol: 170.0 μg/mL, reference: 4.3 – 176.0 μg/mL). However, subsequent ACTH measurements showed a slight elevation (serum ACTH: 66 pg/mL, reference: 0 – 46 pg/mL). As there were no clinical features suggesting hypercortisolemia the cause for symptoms described above was interpreted as sustained stress and intense chronic exercise. In 2016 the patient experienced a progression in weight gain, depression, and mood swings. As a next step, an overnight 1 mg *Dexamethasone* test was done where no suppression was reached with a cortisol level of 546 nmol/L which raised the suspicions for Cushing’s disease. Following this high dose two-day as Dexamethasone test was advised. Test results showed suppression in cortisol levels (**Supplementary Table 3.2**).

**Figure 2:**
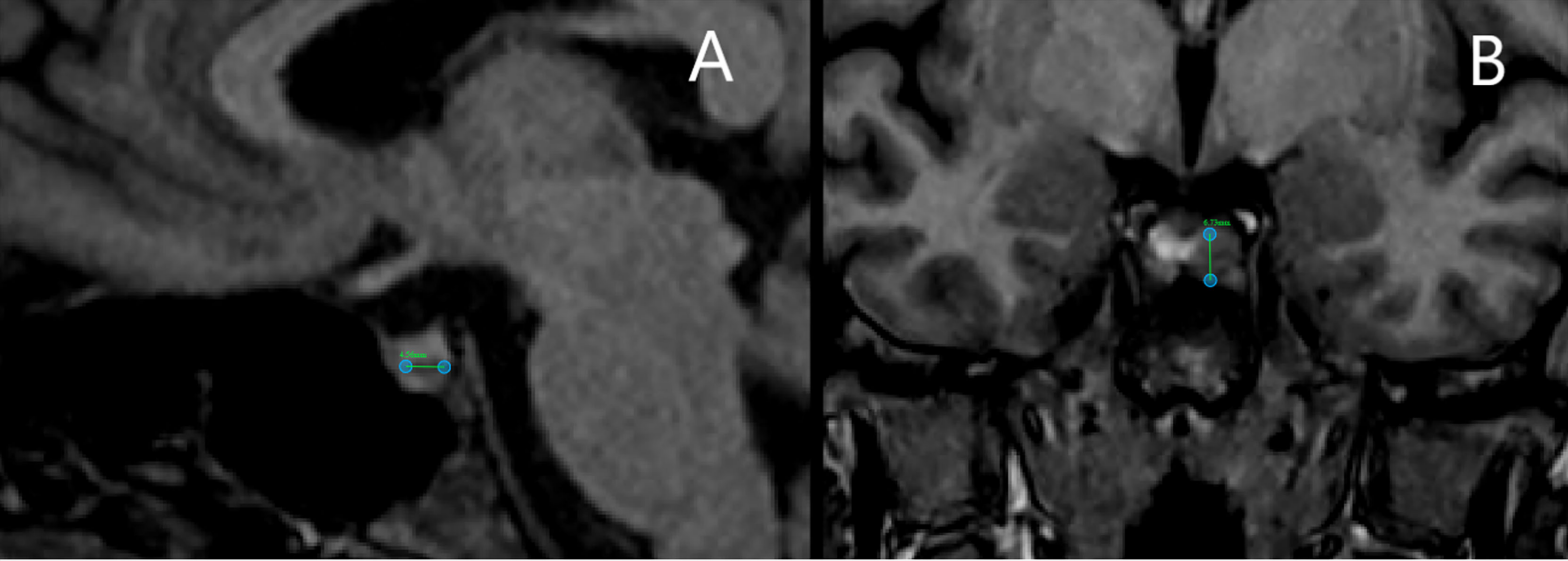
Magnetic resonance imaging scans of bilateral inferior petrosal sinus sampling case patient. **(A)** Scan before bilateral inferior petrosal sinus sampling shows a pituitary tumor of 4.6 mm in diameter. **(B)** Scan shortly before surgery shows an increase in tumor volume with a diameter of 6.7 mm.

In 2018 the patient underwent dynamic gadolinium-enhanced MRI of the sellar region (**Figure 2A**) which demonstrated left-sided microadenoma (0.46 cm in diameter) at the anterior pituitary. To confirm that the PitNET is ACTH secreting additional *Dexamethasone* suppression tests were done (**Supplementary Table 3.3**). According to the laboratory, radiologic and clinical findings, there was an ambiguity concerning low ACTH levels (**Supplementary Table 3.3**). Consequently, a multidisciplinary team of endocrinologists and radiologists decided to perform BIPSS, to substantiate the source of ACTH as PitNET or ectopic. Elevated central/peripheral ACTH ratio was achieved both before and after hCRH stimulation from the sinistral side, as a result, CS of PitNET origin was confirmed (**Table 1**). Repeated MRI shortly before surgery showed an increase in tumor volume (**Figure 2B**).

### MiRNA Annotation and Expression Profiles in BIPSS and PVB Plasma

**Figure 3:**
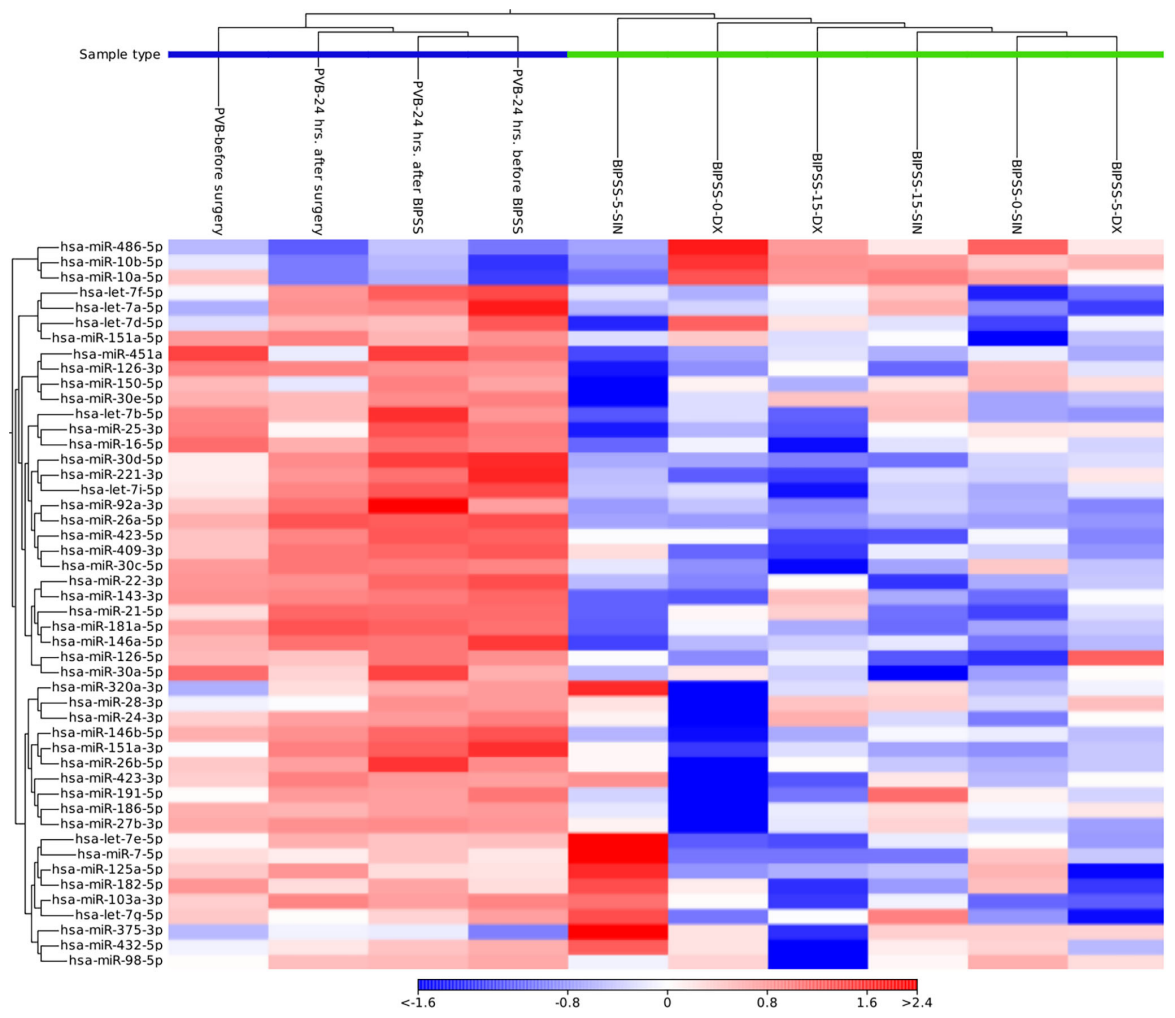
Heat map containing expression patterns of miRNAs to differentiate plasma collected from BIPSS vs PVB. The heat map was generated by selecting a fixed number of features (50) with the highest coefficient of variation and with a minimum supporting count of 1000 reads. Distance measure - Euclidean distance. Cluster analysis showed that there is a clear difference in the expression of miRNAs between BIPSS and PVB samples.

Annotation to miRBase (release v22) for BIPSS samples on average had the annotated reads percentage of - 2.30% (60’884 reads), (range: 1.85 - 3.16%, 41’058 - 95’297 reads). For PVB samples the average annotated read count was 10.98% (336’517 reads) (9.36 - 15.33%, 202’297 - 445’487 reads) (**Supplementary Table 4**). We compared miRNA expression patterns of all 10 plasma samples and identified 50 miRNAs whose expression profiles were able to distinguish PVB plasma vs plasma samples from BIPSS (**Figure 3**). In the clustering analysis, two clusters of samples can be observed: 1^st^ one which represents the plasma from BIPSS containing all six BIPSS samples, and 2^nd^ one which represents the plasma acquired from PVB containing all 4 PVB samples. The expression profile of the 0 min dextral sample was closer to the 15 min dextral sample than to the 5 min sample. Interestingly, the expression profile of 5 the min sinistral sampleshows a high upregulation of several miRNAs compared to other BIPSS samples.

### Differentially Expressed MiRNAs Between Sinistral vs Dextral Side

**Figure 4:**
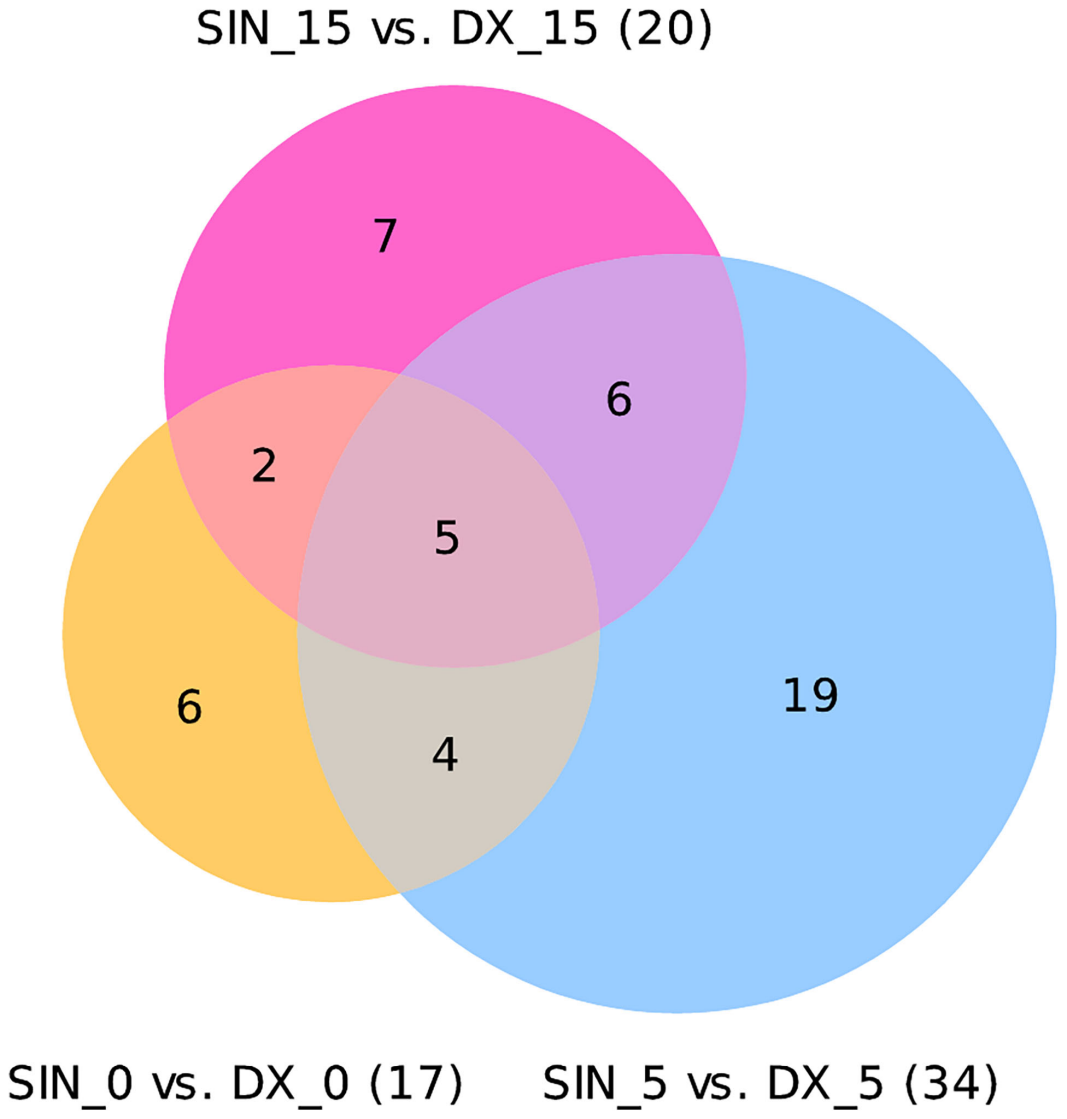
Venn diagram of differentially expressed miRNAs in three time points. The Venn diagram represents differentially expressed miRNAs with a Bonferroni P < 0.05. In a time point of 0 minutes 17 DEMs were found 17 DEMs (12 were upregulated, 5 downregulated). In a time point of 5 minutes 34 DEMs were found (20 upregulated,14 downregulated). In a time point of 15 minutes 20 DEMs were found (11 upregulated, 9 downregulated).

**Figure 5:**
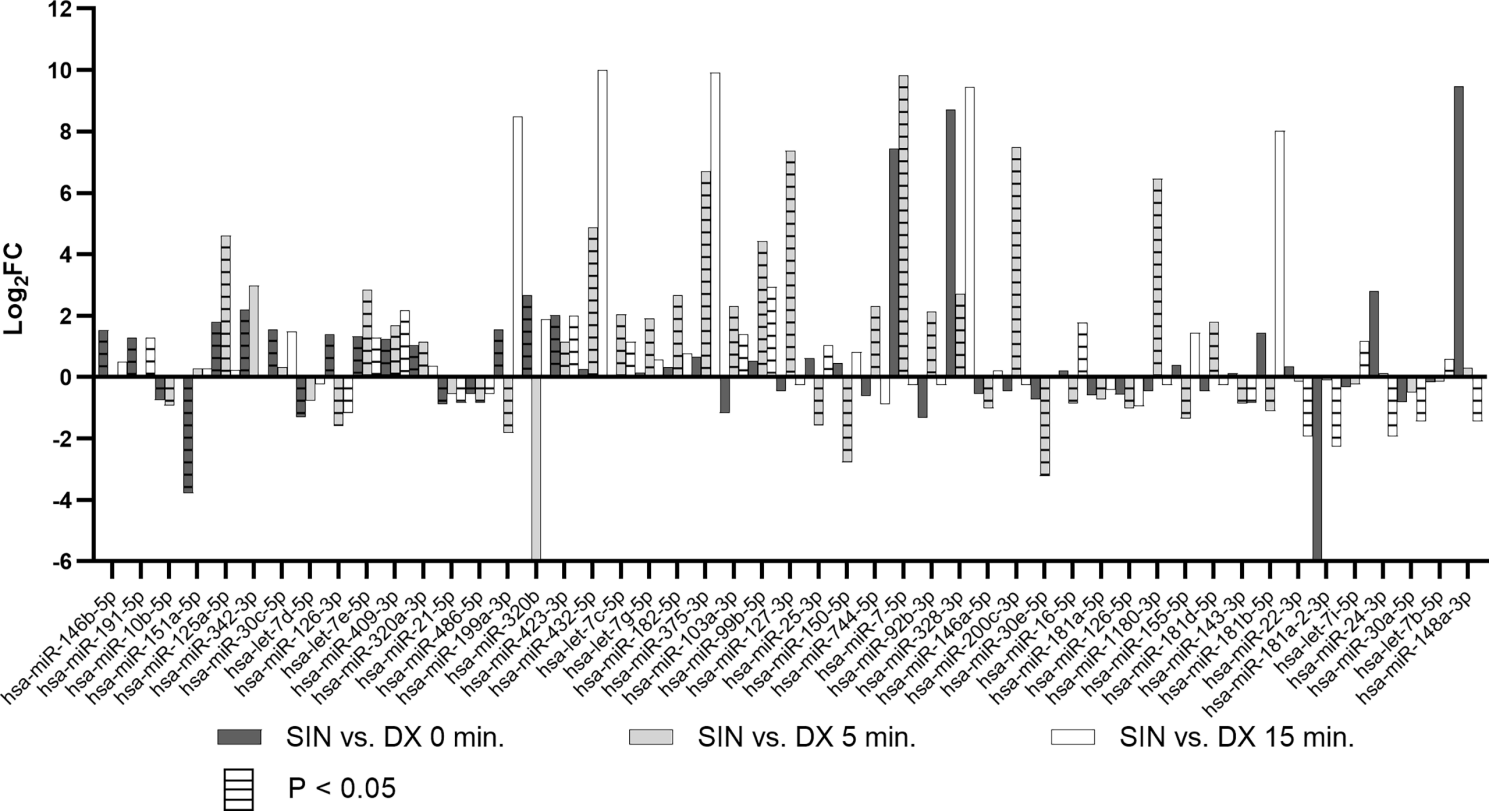
Differentially expressed miRNAs in BIPSS samples across all three time points. The bar chart represents Log2FCs of all differentially expressed miRNAs with Bonferroni P < 0.05. Bars without horizontal lines represent fold changes of adjacent time points (0, 5, 15) but are not statistically significant (Bonferroni P > 0.05). DX - dextral side, SIN - sinistral side.

Analyzing all three time points of BIPSS we identified 49 unique differentially expressed miRNAs (DEMs) between the sinistral and dextral sides (**Figure 4**). Of these 12 DEMs were repeatedly found in two time points and 32 in only one time point. Five DEMs that were repeatedly differentially expressed across all three time points were: hsa-miR-7e-5p, hsa-miR-423-3p, hsa-miR-486-5p, hsa-miR-409-3p, hsa-miR-126-3p. By examining all three time points together a dynamic can be seen which shows that the highest number of DEMs is present during the 5^th^ minute of BIPSS. The highest Log2FC values were also present within the 5^th^ minute, and they ranged from -3.2 to 9.8. In comparison before administration of CRH (0 min), the Log2FC values ranged from -3.8 to 2.7 and during the 15^th^ minute after CRH administration the values ranged from -2.3 to 2.9 (**Figure 5**), (for more detailed information see **Supplementary Table 6**).

During the 5^th^ minute of BIPSS, we detected two DEMs of interest: hsa-miR-375-3p (Log2FC: 6.7, 696.9 CPM 5^th^ min dextral vs 72625.89 CPM 5^th^ min sinistral side) and hsa-miR-7-5p which had the highest Log2FC of all DEMs (9.8, 21.8, CPM 5^th^ min dextral vs 20340.3 CPM 5^th^ min sinistral side). These two DEMs were also upregulated in peripheral plasma 24 hrs. after BIPSS with a Log2FC for hsa-miR-375-3p: 3.3 (20.4 CPM before BIPSS vs 208.8 CPM after BIPSS) and hsa-miR-7-5p: 1.1 (120.6 CPM before BIPSS vs 251.8 CPM after BIPSS). They were not differentially expressed in any of the other BIPSS time points.

### RT-qPCR Testing of MiR-7-5p and MiR-375-3p in Pre- and Postoperative Plasma Samples of ACTH Secreting PitNET Patients

**Figure 6:**
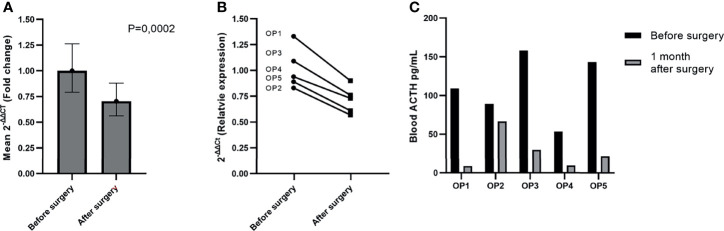
Reduction of miR-7-5p in plasma 24 hours after surgery. **(A)** Graph represents the expression values were calculated with ΔΔCt method and are represented as fold change. In our sample set of five patients miR-7-5p was slightly downregulated in plasma 24 hours after surgery (P = 0.0002). **(B)** Graph represents the relative expression of miR-7-5p before and after surgery in each patient individually. The P value was calculated using paired t-test. **(C)** Graph represents ACTH measurements taken shortly before and one month after the PitNET surgery.

The NGS results showed that miR-375-3p and miR-7-5p are secreted within sinistral IPS (tumor side) in vastly higher amounts than in left IPS upon stimulation by hCRH. This indicates that these two miRNAs are potentially of ACTH secreting PitNET origin. Therefore, we hypothesized that there should be a slight downregulation of these miRNAs in peripheral plasma 24 hours after resection of PitNET. To test this hypothesis, we employed RT-qPCR. The expression of miR-375-3p and miR-7-5p was analyzed in a sample set of 5 patients (**Table 1**) from which plasma samples were taken before surgery and 24 hours after surgery. The RT-qPCR results show that there is a slight decrease in levels of miR-7-5p in patients’ plasma 24 hours after surgery (P = 0.0002) (**Figure 6A**). As for the miR-375-3p it was detected in only one out of 9 plasma samples, therefore, its expression could not be measured. By analyzing each patient individually, a slight downregulation of miR-7-5p in peripheral plasma 24 hours after surgery can be observed for all patients (**Figure 6B**). We also measured ACTH levels in PVB before surgery and one month after surgery and a decrease in ACTH levels was observed for all patients (**Figure 6C**).

### Expression of MiR-7-5p and MiR-375-3p in Tumor Samples

**Figure 7:**
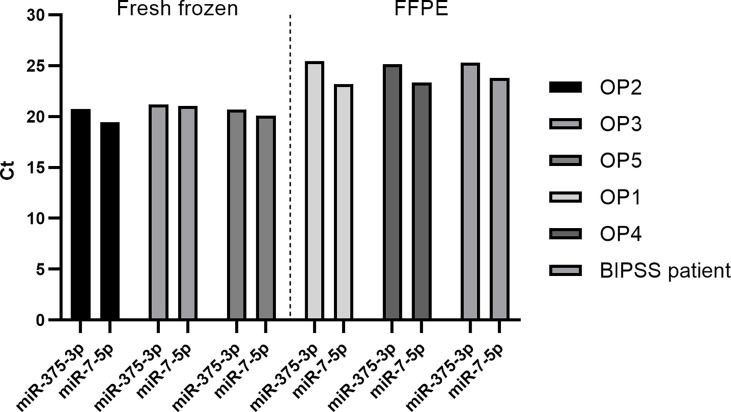
Expression of miR-7-5p and miR-375-3p in ACTH secreting PitNET tissue samples. The expression is represented by Ct values on Y axis. OP2, OP3 and OP5 belong to fresh frozen tissue samples group while OP1, OP4 and BIPSS patient belong to FFPE samples group. The expression of both miRNAs is stable within ACTH secreting PitNET tissues. There is a difference in Ct values between FFPE samples and fresh frozen samples.

We also validated the expression of miR-7-5p and miR-375-3p within tissue samples of our pre- and post-operative patient cohort consisting of five ACTH secreting PitNET patients. Additionally, we checked the expression of these markers within the BIPSS patient’s tumor sample as well. Due to sample availability for some patients, the RNA was extracted from fresh frozen tissue samples, and for other patients, it was extracted from FFPE samples. The RT-qPCR data shows that both miR-375-3p and miR-7-5p are stably expressed in all tissue samples (**Figure 7**). However, there are some differences in levels of miR-375-3p and miR-7-5p between fresh-frozen and FFPE tissue samples which can be explained by RNA degradation due to the fixation process and different RNA extraction methods used. These results confirm that the ACTH secreting PitNETs express both miR-375-3p and miR-7-5p.

### Expression of MiR-7-5p in ACTH Secreting PitNETs vs. NF PiNETs

**Figure 8:**
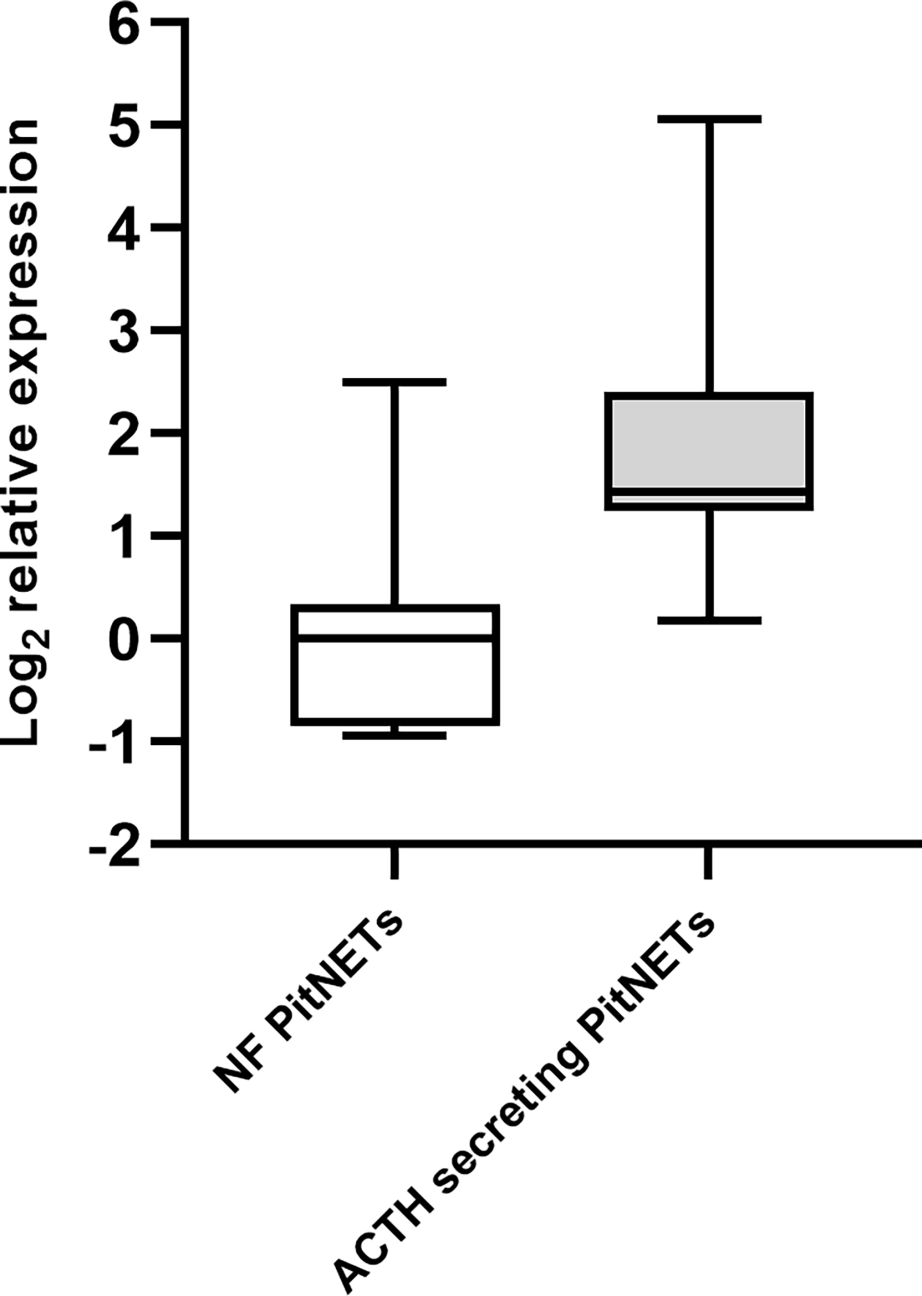
Expression of miR-7-5p in ACTH secreting PitNET patients vs. NF-PitNET patients. The box plot represents the differences in Log_2_ relative expression of miR-7-5p in ACTH secreting PitNET samples (n = 12) vs. NF-PitNET patients (n = 9). The results show that miR-7-5p is significantly upregulated in ACTH secreting PitNET patients plasma (P = 0.0013). The P value was calculated using Wilcoxon rank sum test.

To further evaluate if the plasma levels miRNA miR-7-5p are influenced by the presence of ACTH secreting PitNET we tested this miRNA in a group of 12 ACTH secreting PitNET patients against a group of 9 NF-PitNET patients. Details on patient characteristics can be found in **Supplementary Table 1**. The RT-qPCR results show that the plasma levels of miR-7-5p are significantly higher in the patients harboring ACTH secreting PitNET (3.5-fold increase) compared to the patients with NF PitNETs (P = 0.0013) (**Figure 8**). Similarly, to pre and postoperative plasma results we could not detect miR-375-3p in most of the samples.

## Discussion

Despite the vast advancements in the research of circulating miRNAs in cancers (22) the studies of circulating plasma miRNAs in PitNETs remain rare with only four published reports (17, 18, 23, 24). The reason for the scarce amount of studies could be that the hormone-secreting PitNETs with clinical manifestations are generally rare in the population (25). Also, they are less proliferative than malignant tumors therefore once the PitNET secreted miRNAs are diluted within the blood their detection becomes difficult requiring highly sensitive methods. To our knowledge, this is the first study that has evaluated the circulating miRNAs in plasma samples that are acquired directly from BIPSS, a “gold standard” procedure in the diagnosis of ACTH secreting PitNETs. As a result, we were able to identify two miRNAs that are potentially PitNET derived.

In BIPSS the location of the sampling catheter is guided *via* a transjugular approach to the IPSs which are in close proximity to the pituitary gland. This allows to precisely assess the amount of secreted ACTH after the gland and PitNET is stimulated with CRH (6). This in theory should increase the possibility to detect PitNET specific circulating miRNAs since their dilution is less than in blood sampled from PVB. More so a recent study published by Gümürdü A. showed for the first time that the stimulation of hormone release *via* exocytosis of large dense-core vesicles (LDCVs) is accompanied by the release of miRNAs. Their data concluded that in bovine chromaffin cells specific miRNAs (in their case miR-375) can be released upon stimulation alongside hormones and neurotransmitters (ribomones) (26).

Our data strongly support the idea of ribomones as we observed a strong increase in plasma expression of two LDCV packed miRNAs hsa-miR-375-3p and hsa-miR-7-5p (**Figure 5**) within the sinistral side during 5 minutes of BIPSS. According to our clinical data, the tumor of the patient was located on the sinistral side and the highest amount of ACTH release was achieved during the 3^rd^ and 5^th^ minute of the procedure which was 24 times higher than in the dextral side (**Table 2**). Also, the amount of hsa-miR-375-3p and hsa-let-7-5p reads in PVB plasma before and after BIPSS was much lower than in the BIPSS-5-SIN sample further suggesting that they have potentially a PitNET origin and are specifically associated with the secretion of ACTH. Since hCRH during the BIPSS is administered peripherally (6) the peripheral sampling of these two miRNAs could potentially be used alternate method asses the presence of ACTH secreting tumor without the need to directly intervene with IPS – a highly invasive procedure.

**Table 2:** Bilateral inferior petrosal sinus sampling ACTH levels.

Minutes after CRH stimulation	Sinus petrosus inferior dextral (ACTH pg/ml)	Sinus petrosus inferior sinistral (ACTH pg/ml)
-5	22.7	277
0*	24	189
3	36.2	777
5*	36.2	552
10	37.1	333
15*	33.8	501

(Bold)* - during these steps the blood samples were also collected for miRNA analysis. CRH, corticotropin releasing hormone, ACTH, adrenocorticotropic hormone.

Decreased levels of miR-7-5p could also be detected by RT-qPCR in five patients plasma samples collected 24 hours after surgery (**Figure 6A, B**). Furthermore, it was found to be upregulated in ACTH secreting PitNET patients’ plasma compared to NF PitNET patients’ plasma (**Figure 8**). We also confirmed that miR-7-5p and miR-375-3p are both stably expressed in ACTH secreting PitNET tissues (**Figure 7**). Overall these results are further suggesting that the plasma levels miR-7-5p could be associated with ACTH secreting PitNETs. However, these results must be interpreted with caution due to the small sample size of five patients in pre and post operative plasma patient group. Interestingly miR-375-3p could not be detected within most of the 17 ACTH secreting patients’ plasma samples despite it being stably expressed within tumor tissues. Perhaps the presence of this miRNA blood might be purely associated with an intravenous administration hCRH which extensively stimulates the exocytosis of ACTH granules. Another reason why this miRNA was not detected by qPCR could be due to variations at 3’ and 5’ end of miRNA sequence (isomiR) (27).

In regards to other tumors, hsa-miR-7-5p has been studied in non-small cell lung cancers, glioblastomas, and nasopharyngeal carcinomas with the majority of studies inclining towards hsa-miR-7-5p having tumor suppressing characteristics (28–30). hsa-miR-375-3p has also been reported in the majority of studies as a tumor-suppressive miRNA in cancers such as colorectal cancers, bladder cancers, and head and neck squamous cell carcinomas (31–33). To further evaluate how these two miRNAs affect the phenotype of ACTH secreting PitNETs functional studies are needed.

A recently published study showed that plasma miRNAs miR-16-5p, miR-145-5p, and let-7g-5p are upregulated in CS patients with ACTH-secreting PitNETs versus CS patients with ectopic cause (18). In our samples, let-7g-5p was upregulated in sinistral side (Log2FC: 1.9) at the 5^th^ minute mark while miR-16-5p was found to be downregulated (Log2FC: -0.9). Interestingly, miR-16-5p was upregulated at the 15^th^ minute time point (Log2FC: 1.8) (**Figure 5**). In our study miR-145-5p was not differentially expressed in any of the time points. Upregulation of miR-16-5p and let-7g-5p within the sinistral side at 15 minutes after stimulation by CRH could indeed indicate that these miRNAs might be of PitNET origin, however functional studies are required to validate this claim. There have also been studies in ACTH secreting PitNET tissues. A study by Amaral et al. compared 14 tumor samples to seven normal pituitaries from autopsies using the Serial analysis of gene expression (SAGE) method and validation by qPCR (34). The results showed a downregulation of eight miRNAs of which let-7a, miR-15a, miR-16, miR-21, miR-143, and miR-150 were also differentially expressed in our BIPSS patient’s NGS dataset (**Supplementary Table 6**) (34). let-7a-5p variant was significantly upregulated in plasma after surgery (Log2FC: 0.5). miR-15a-5p was downregulated in plasma after surgery (Log2FC: -1.1). miR-16-5p was downregulated in the the sinistral side during 5^th^ minute of BIPSS (Log2FC: -0.9) however it was upregulated in the sinistral side during the 15^th^ minute (Log2FC: 1.8). miR-21-5p was downregulated in the sinistral side at 0 minutes and 15^th^ minute of BIPSS (Log2FC: 0.9 and 0.88). miR-143-3p was downregulated in the sinistral side at 5^th^ minute and 15^th^ minute time points of BIPSS (Log2FC: 0.9 and 0.8). miR-150-5p was downregulated in the sinistral side at the 5^th^ minute mark of BIPSS and in plasma after surgery (Log2FC: -2.8 and -0.7). The downregulation of these miRNAs in the sinistral plasma (tumor side) shows that these results somewhat overlap with the reported results of Amaral et al. (34) as they were also downregulated within the tumor compared to normal pituitary tissues.

In conclusion, this study has provided the first insight into plasma miRNome from samples that are acquired from the BIPSS procedure allowing us to investigate PitNET secreted miRNAs in close proximity to the tumor itself. We were able to distinguish BIPSS sample miRNA composition from PVB fraction. Furthermore, the results indicate that analysis of samples taken directly from IPS are a promising source for ACTH secreting PitNET associated plasma biomarker discovery as the levels hsa-miR-7-5p and hsa-miR-375-3p rapidly increased within sinistral IPS (tumor side) upon the stimulation by CRH. Further RT-qPCR testing of miR-7-5p in PVB plasma confirmed that this miRNA can potentially serve as biomarker for blood-based assay development to diagnose ACTH secreting PitNETs.

## Data Availability Statement

Data supporting the findings that are reported within the manuscript are available in **Supplementary Material**. Sequencing data are available in FASTQ file format at Gene Expression Omnibus public repository with accession number: GSE163957. Link: https://www.ncbi.nlm.nih.gov/geo/query/acc.cgi?acc=GSE163957. Any additional data is available from corresponding authors upon request.

## Ethics Statement

The studies involving human participants were reviewed and approved by Central Medical Ethics Committee of Latvia (protocol: 22.03.07/A7 and 2/18-02-21). The patients/participants provided their written informed consent to participate in this study. Written informed consent was obtained from the individual(s) for the publication of any potentially identifiable images or data included in this article.

## Author Contributions

HN performed RNA extraction, library preparation, NGS data analysis. Manuscript was prepared by HN, RP, and VR. Patients clinical data was provided by IK and MR. All the figures and tables within manuscript and supplementary were prepared by HN and reviewed by RP and VR. Patient management and recruitment was done by IK, IB, MR, LS, JSt, and JSo. All authors contributed to the article and approved the submitted version.

## Funding

This research was funded by the European Regional Development Fund within the project RNA molecular determinants in development of pituitary adenoma” (1.1.1.1/18/A/089).

## Conflict of Interest

The authors declare that the research was conducted in the absence of any commercial or financial relationships that could be construed as a potential conflict of interest.

## Publisher’s Note

All claims expressed in this article are solely those of the authors and do not necessarily represent those of their affiliated organizations, or those of the publisher, the editors and the reviewers. Any product that may be evaluated in this article, or claim that may be made by its manufacturer, is not guaranteed or endorsed by the publisher.
